# Health state preferences associated with weight status in children and adolescents

**DOI:** 10.1186/1471-2431-11-12

**Published:** 2011-02-07

**Authors:** Mandy B Belfort, John AF Zupancic, Katherine M Riera, Jane HG Turner, Lisa A Prosser

**Affiliations:** 1 Div of Newborn Medicine, Children's Hospital Boston/Harvard Medical School, Boston MA, USA; 2 Dept of Neonatology, Beth Israel Deaconess Medical Center/Harvard Medical School, Boston MA, USA; 3 Child Health Evaluation and Research Unit, Div of General Pediatrics, U. of Michigan, Ann Arbor MI, USA

## Abstract

**Background:**

Childhood obesity is a substantial public health problem. The extent to which health state preferences (utilities) are related to a child's weight status has not been reported. The aims of this study were (1) to use a generic health state classification system to measure health related quality of life and calculate health utilities in a convenience sample of children and adolescents and (2) to determine the extent to which these measures are associated with weight status and body mass index (BMI).

**Methods:**

We enrolled 76 children 5-18 years of age from a primary care clinic and an obesity clinic in Boston MA. We administered the Health Utilities Index (HUI) and used the HUI Mark 3 single- and multi-attribute utility functions to calculate health utilities. We determined BMI percentile and weight status based on CDC references. We examined single-attribute and overall utilities in relation to weight status and BMI.

**Results:**

Mean (range) age was 10.8 (5-18) years. Mean (SD) BMI percentile was 76 (26); 55% of children were overweight or obese. The mean (SD) overall utility was 0.79 (0.17) in the entire sample. For healthy-weight children, the mean overall utility was higher than for overweight or obese children (0.81 vs. 0.78), but the difference was not statistically significant (difference 0.04, 95% CI -0.04, 0.11).

**Conclusions:**

Our results provide a quantitative estimate of the health utility associated with overweight and obesity in children, and will be helpful to researchers performing cost effectiveness analyses of interventions to prevent and/or treat childhood obesity.

## Background

Childhood obesity is a substantial and growing public health problem [1] and numerous interventions for its treatment and prevention have been developed [2,3]. In deciding which interventions are most efficient, cost-effectiveness analysis can be used to compare the intervention-associated costs with the benefits, including improvements in health status [4]. Previous research has demonstrated that in children and adolescents, higher body mass index (BMI) is associated with lower health related quality of life (HRQOL) [5-9], suggesting that preventing or treating obesity would improve children's HRQOL. While the HRQOL measures used in those studies are useful for describing health status, they are not applicable to cost-effectiveness analysis because they do not reflect the value attached to the health status, either by the participants or by society.

One well-accepted economic method for quantifying people's value for health is to measure health preferences or utilities, numerical scores that represent the value an individual assigns to a particular health state, with 1 representing full health and 0 representing death [10]. In contrast to HRQOL or health status, which describe particular health states, health utilities reflect the value or preference given to the state of health. Obesity-related health utilities have been published for adults [11-14], but not for children or adolescents. Estimating health utilities in the pediatric population would be useful for researchers studying childhood obesity treatment and prevention interventions, and would allow direct economic comparison of obesity-related intervention strategies with each other, and with interventions for other diseases.

Certain generic HRQOL survey instruments allow the classification of health status as well as calculation of health utilities associated with the health status. The Health Utilities Index (HUI) [15] is one such instrument that has been used extensively in children and adolescents, but has not been studied in relation to child and adolescent obesity. The aims of our study were (1) to use a generic health state classification system to collect pilot data regarding HRQOL and calculate health utilities related to overweight and obesity in a convenience sample of children and adolescents seeking primary or obesity-related health care at an academic children's hospital, and (2) to compare the self-rated health status to health status as reported by proxy (a parent).

## Methods

### Study design and participants

For this cross-sectional survey, we recruited children 5-18 years of age who were attending well-child appointments at an academic children's hospital-based primary care clinic in Boston, MA (n = 72), and children attending a specialty obesity clinic located in the same clinical area (n = 4). We excluded families seeking care for acute medical conditions, and families who could not complete the study questionnaires in English. Study staff provided eligible families with a letter that described the study. Consent was obtained when the parent and child verbally agreed to complete the study interview. The Children's Hospital Boston human subjects committee approved the study protocol.

### Measurements

#### Health status and utilities

To measure health status, we used the Health Utilities Index ( HUI, Health Utilities Inc., Dundas ON, Canada) [15], a 40-item interviewer-administered questionnaire. The HUI is a generic measure of health status that has been used extensively in both clinical and general populations, including children [16]. The HUI questionnaire asks about functioning in each of the following areas: vision, hearing, speech, ambulation, dexterity, self-care, emotion, memory, thinking, and pain/discomfort.

Using the Mark 3 scoring algorithm [17], responses are converted to single-attribute utility scores, which reflect the level of functioning in the following domains: vision, hearing, speech, ambulation, dexterity, emotion, cognition, and pain; and an overall multi-attribute utility score, which incorporates all domains and reflects the participants' overall health status. The Mark 2 scoring algorithm [18] uses responses to the same questionnaire to generate single-attribute utility scores for the following domains: sensation, mobility, emotion, cognition, self-care, and pain; and an overall multi-attribute utility score.

An advantage of the HUI over many other instruments that measure health status in children is that, in addition to measuring health status, the HUI provides a weighting algorithm to calculate health utilities. While health status is a description of a particular state of health, health utilities reflect the value or preference given to the state of health. A health utility is a single summary score that incorporates all positive and negative aspects of the health state. Single attribute utility scores range from 0 (most disabled) to 1 (non-disabled). For multi-attribute utility scores, 0 represents death and 1 represents perfect health; scores less than 0 are also possible and represent health states considered to be worse than death. The HUI algorithm assigns utilities to health states based on preferences elicited from a large community sample [17].

The HUI is recommended for children age 5 years of age and older, and can be given as a self-assessment (8 years and older) and/or by proxy, such as a parent, who answers questions on behalf of the participant (5 years and older). Proxy respondents are used commonly for young children and others who, due to cognitive limitations, cannot respond reliably to the questionnaire. We administered the proxy version of the HUI to a parent of all participants. For participants 8 years of age or older, we also administered the HUI self-assessment directly to the child [19]. Questions were asked regarding current health "in the past 4 weeks."

#### Anthropometry

As part of routine clinical care, nurses or medical assistants weighed participants with a regularly calibrated digital scale (Scale-tronix model 6002, White Plains NY) and measured them with a stadiometer (Perspective Enterprises, Portage MI). From the electronic medical record, we abstracted measurements obtained the same day that participants completed study questionnaires, as well as the calculated BMI (kg/m^2^).

#### Medical history and sociodemographic information

Parents completed a short questionnaire regarding the child's medical history including diagnoses and medications, and sociodemographic information about the family.

### Analysis

For our main analyses, we used the HUI Mark-3 (HUI3) single- and multi-attribute utility functions [17] to calculate utility values scaled from 0 = dead to 1 = perfect health, with values less than 0 representing health states considered to be worse than death. We focused our analyses on the overall utility, as well as single-attribute utilities relating to ambulation, emotion, cognition, and pain, which we believed to be most relevant to overweight/obesity. Other domains in the HUI include vision, hearing, speech, and dexterity. We performed secondary analyses using the HUI Mark-2 (HUI2) utility functions [18] which use responses from the same 40-item questionnaire to generate single-attribute utilities for sensation, mobility, self care, emotion, cognition, and pain as well as an overall utility value. We used Centers for Disease Control and Prevention guidelines to define weight status as healthy (BMI less than the 85th percentile for age) and overweight or obese (BMI greater than or equal to the 85^th ^percentile).

We first examined correlations of self- and proxy-reported utility values by calculating Spearman correlation coefficients and p-values. Next, we calculated median and mean utility values in each weight status group (healthy weight and overweight/obese) and the difference between means. Due to the non-normal distribution of the utility data, we used bootstrapping [20] to obtain 95% confidence intervals around means and difference between means. Adjusting for age and demographic factors, we used tobit regression to model the association of BMI with overall utility, accounting for the truncated nature of our utility data. Additionally, we compared the frequency of utility values <0.9 between weight status groups. We performed primary analyses in the entire sample, using proxy responses for participants younger than 8 years old and self responses for participants 8 years of age or older. We also performed additional analyses restricted to participants 8 years or older, and 12 years or older, using both self- and proxy assessments.

## Results

In the overall sample, mean (standard deviation, SD) age was 10.8 (3.3) years with a range of 5-18 years. Mean (SD) BMI percentile for age was 76 (26) and 55% of participants were overweight or obese. Table 1 shows characteristics of the study participants according to weight status. Compared with healthy weight children, overweight or obese children appear more likely to be male (57% vs. 47%), more likely to have asthma (33% vs. 18%), and less likely to be white (7% vs. 18%).

**Table 1:** Characteristics of 76 participants by weight status*

	Healthy weight n = 34	Overweight or obese n = 42
**Child**	**Mean (SD), range**	

Age (years)	11.5 (3.6), 5-18	10.3 (2.9), 5-17

Weight-for-age percentile	55 (19), 16-83	94 (7.4), 72-100

Height-for-age percentile	57 (27), 1-98	69 (31), 9-100

BMI-for-age percentile	52 (22), 6-84	96 (4), 85-100

	**Number (%)**	

Male	16 (47)	24 (57)

Medical conditions:		

Asthma	6 (18)	14 (33)

High blood pressure	1 (3)	3 (7)

High cholesterol	2 (6)	1 (2)

Other	6 (18)	4 (10)

Takes regular medication	9 (26)	13 (31)

**Parent**		

Ever told by provider that child is overweight or obese	1 (3)	28 (67)

Household income		

<$20,000	9 (26)	16 (38)

$20,000-$60,000	19 (56)	16 (38)

>$60,000	5 (15)	9 (22)

Don't know/missing	1 (3)	1 (2)

Education		

HS diploma or less	10 (29)	13 (31)

Some college	24 (71)	26 (62)

BA, BS, grad, or professional	0	3 (7)

Race/ethnicity		

White, non-Hispanic	6 (18)	3 (7)

Black, non-Hispanic	18 (53)	22 (52)

Hispanic	10 (29)	12 (29)

Asian/Mixed/Other	0	5 (12)

*According to Centers for Disease Control and Prevention standards

**Figure 1:**
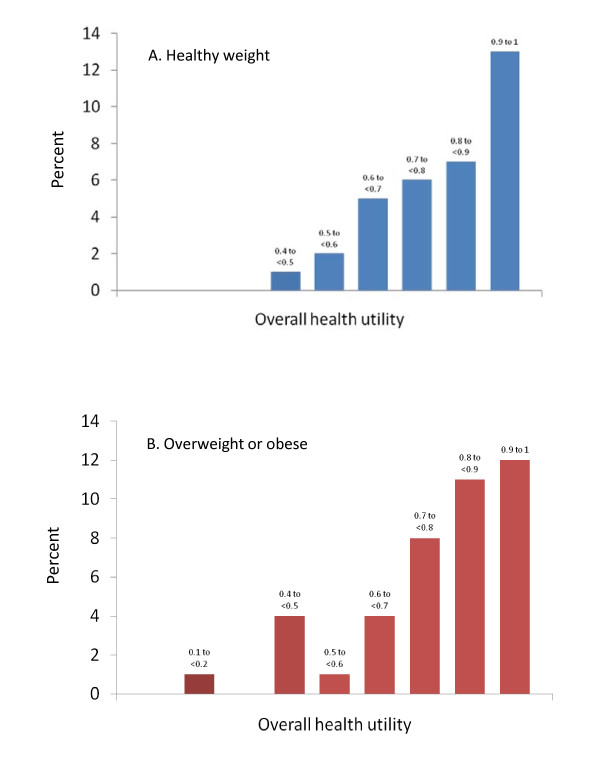
**Frequency distribution of overall health utilities in (A) 34 healthy weight and (B) 41 overweight/obese study participants**. Utilities are scaled to values of 0 = death and 1 = perfect health. For participants ≥8 years of age, utilities are calculated from the HUI self assessment and for participants <8 years, from the HUI proxy assessment.

The mean (SD) overall HUI3 utility score was 0.79 (0.17) and the mean single-attribute HUI3 utility values ranged from 0.87 for cognition to 1.0 for ambulation (Table 2). Figure 1 shows the frequency distribution for overall HUI3 utility values.

**Table 2:** Selected single-attribute and overall health utility values for 76 participants*

	Median	Mean (SD)	Range
Ambulation	1.0	1.0 (0.02)	0.83-1.0

Emotion	1.0	0.94 (0.12)	0.33-1.0

Cognition	0.92	0.87 (0.16)	0.32-1.0

Pain	1.0	0.91 (0.18)	0-1.0

**Overall**	**0.86**	**0.79 (0.17)**	**0.17-1.0**

*HUI3 by proxy for <8 y.o. and by self-assessment for ≥8 y.o.

Correlations of HUI3 utilities calculated from self-assessment questionnaires and proxy questionnaires (Table 3) were modest, ranging from 0.14 (p = 0.30) for pain to 0.47 (p = 0.0003) for overall utility. We calculated these correlations only for participants 8 years of age or older, because we did not administer the HUI self-assessment questionnaire to children younger than 8 years old. For ambulation, all but one participant's self- and proxy-assessed utilities were equal to 1, so a Spearman correlation could not be calculated.

**Table 3:** Spearman correlations and p-values for selected self-reported and proxy health utility values for 63 participants ≥8 years old

	*Self-reported*				
		Emotion	Cognition	Pain	Overall

	Emotion	**0.45** **p = 0.0003**	0.17 p = 0.21	0.38 p = 0.003	0.38 p = 0.003

***Parent-reported***	Cognition	0.29 p = 0.04	**0.30** **p = 0.02**	0.13 p = 0.33	0.36 p = 0.005

	Pain	0.10 p = 0.44	0.07 p = 0.61	**0.14** **p = 0.30**	0.19 p = 0.15

	Overall	0.46 p = 0.0003	0.28 p = 0.03	0.37 p = 0.004	**0.47** **p = 0.0002**

Comparing the overall HUI3 utility values between healthy weight and overweight/obese participants in the entire sample (Table 4), both the median and mean utility values for healthy weight participants were somewhat higher (mean difference 0.04, 95% CI -0.04, 0.12). For the single-attribute HUI3 utility values emotion, cognition, and pain, the mean values for healthy weight participants were also slightly higher than for overweight/obese participants. Mean values for the other domains - vision, speech, hearing, and dexterity - were similar between the groups (data not shown). We also categorized overall and single-attribute utility values as less than or greater than 0.9, and found that 62% of healthy weight participants and 71% of overweight/obese participants had overall utility HUI3 values <0.9. Results were similar for single-attribute utility values (data not shown). Adjusting for age, sex, maternal education, race/ethnicity, and annual household income, for each additional 10 BMI units, overall HUI3 utility decreased by 0.04 (95% CI -0.11, 0.03). None of the demographic factors had an independent association with health utility.

**Table 4:** Single attribute and overall preference values by weight status

	*Healthy weight*		*Overweight or obese*		*Difference*	
**All participants (n = 76)**						

	***Median (IQR)***	***Mean (95% CI)***	***Median (IQR)***	***Mean (95% CI)***	***Difference in means***	***95% CI***

Ambulation	1 (1,1)	1 (1,1)	1.0 (1,1)	1.0 (0.99, 1)	0	0, 0.01

Emotion	1 (0.91, 1)	0.95 (0.90, 0.98)	1 (0.91, 1)	0.94 (0.90, 0.97)	0.01	-0.05, 0.06

Cognition	0.92 (0.86, 1)	0.87 (0.82, 0.92)	0.92 (0.86, 1)	0.86 (0.81, 0.91)	0.01	-0.06, 0.08

Pain	1 (0.92, 1)	0.94 (0.89, 0.98)	1 (0.77, 1)	0.88 (0.82, 0.94)	0.05	-0.02, 0.13

**Overall**	**0.88 (0.71, 0.93)**	**0.81 (0.76, 0.86)**	**0.83 (0.70, 0.93)**	**0.78 (0.72, 0.83)**	**0.04**	**-0.04, 0.11**

**Participants ≥8 years old (n = 63)**						

*Self-assessment*						

Ambulation	1 (1,1)	1 (1,1)	1 (1, 1)	1 (1,1)	0	0, 0

Emotion	1 (0.91, 1)	0.96 (0.93, 0.98)	1 (0.91, 1)	0.93 (0.88, 0.97)	0.03	-0.02, 0.08

Cognition	0.92 (0.78, 1)	0.87 (0.81, 0.92)	0.92 (0.86, 1)	0.86 (0.81, 0.91)	0.00	-0.07, 0.08

Pain	1 (0.92, 1)	0.95 (0.90, 0.99)	1 (0.77, 1)	0.88 (0.80, 0.95)	0.07	-0.01, 0.16

**Overall**	**0.89 (0.72, 1)**	**0.82 (0.77, 0.88)**	**0.83 (0.63, 0.93)**	**0.77 (0.70, 0.83)**	**0.05**	**-0.03, 0.14**

*Proxy assessment*						
Ambulation	1 (1,1)	1 (1,1)	1 (1, 1)	0.99 (0.97, 1)	0.01	0, 0.03

Emotion	1 (0.91, 1)	0.96 (0.93, 0.99)	1 (0.91, 1)	0.95 (0.91, 0.97)	0.01	-0.03, 0.06

Cognition	0.88 (0.92, 1)	0.88 (0.80, 0.95)	1 (1, 1)	0.96 (0.92, 0.98)	-0.07	-0.16, 0.01

Pain	1 (0.92, 1)	0.91 (0.82, 0.97)	1 (0.92, 1)	0.91 (0.86, 0.96)	0.00	-0.11, 0.09

**Overall**	**0.93 (0.77, 1)**	**0.84 (0.74, 0.92)**	**0.93 (0.75, 1)**	**0.86 (0.82, 0.91)**	**-0.02**	**-0.13, 0.07**

**Participants ≥12 years old (n = 33)**						

*Self-assessment*						

Ambulation	1 (1,1)	1 (1,1)	1 (1,1)	1 (1,1)	0	0, 0

Emotion	1 (1, 1)	0.98 (0.96, 1.0)	1 (0.91, 1)	0.96 (0.91, 0.99)	0.03	-0.01, 0.07

Cognition	0.92 (0.70, 0.92)	0.87 (0.81, 0.92)	0.92 (0.70, 1)	0.84 (0.73, 0.93)	0.02	-0.08, 0.14

Pain	1 (0.92, 1)	0.96 (0.93, 0.99)	1 (0.92, 1)	0.92 (0.84, 0.99)	0.04	-0.04, 0.13

**Overall**	**0.89 (0.72, 0.93)**	**0.85 (0.80, 0.90)**	**0.83 (0.74, 0.93)**	**0.80 (0.70, 0.89)**	**0.05**	**-0.05, 0.16**

*Proxy assessment*						

Ambulation	1 (1,1)	1 (1,1)	1 (1,1)	1 (1,1)	0	0, 0

Emotion	1 (0.96, 1)	0.97 (0.93, 0.99)	1 (0.91, 1)	0.96 (0.92, 0.99)	0.00	-0.04, 0.06

Cognition	1 (0.92, 1)	0.91 (0.81, 0.98)	1 (1,1)	1 (1,1)	-0.10	-0.19, 0.02

Pain	1 (0.92, 1)	0.90 (0.76, 0.99)	1 (0.77, 1)	0.89 (0.78, 0.98)	0.01	-0.15, 0.15

**Overall**	**0.94 (0.81, 1)**	**0.86 (0.73, 0.95)**	**0.97 (0.80, 1)**	**0.90 (0.83, 0.97)**	**-0.05**	**-0.19, 0.08**

IQR is interquartile range and CI is the bootstrapped 95% confidence interval.

We performed additional analyses in participants 8 years of age and older using HUI3 utilities calculated both from self- and proxy-assessments (Table 4). For the overall utility, healthy weight participants rated themselves higher than overweight/obese participants (0.82 vs. 0.77, difference 0.05, 95% CI -0.03, 0.14). In contrast, parents of healthy weight participants did not rate their children higher than parents of overweight/obese parents (0.84 vs. 0.86, difference -0.02, 95% CI -0.13, 0.07). We observed similar trends when we restricted our analyses to participants 12 years of age or older.

Similar to the HUI3, in secondary analyses using HUI2 utilities, we found that for children 8 years and older, the overall utility trended higher in healthy weight participants compared with overweight/obese participants (0.89 vs. 0.85, difference 0.04, 95% CI -0.02, 0.09).

We also used our estimates for the mean overall utilities and SD's in the normal weight and overweight/obese groups to calculate the sample size needed to detect the difference in utility that we observed, assuming a normal distribution [21]. At an alpha level of 0.05 and power 0.8, the necessary sample size would be 182 participants in each group.

## Discussion

In this study, we used a generic heath state classification system to collect pilot data on health status and calculate health utility values in a convenience sample of healthy weight and overweight/obese children and adolescents. As expected, we observed a somewhat higher overall utility value among healthy weight participants. Although our results did not reach statistical significance, our aim was to provide a quantitative estimate of the decrease in utility associated with overweight and obesity status in childhood, as well as the degree of uncertainty around that estimate.

While health status describes a particular state of health, the utility value associated with a health state represents the value placed by the individual - or by society - on that health state, and is used to calculate the quality adjusted life year (QALY), an important component of cost-effectiveness analysis. A valid estimate of the health utility value associated with overweight/obesity is important in the area of obesity prevention and treatment given the large number of interventions being developed in response to the obesity epidemic, and the need to prioritize resources for the most efficient interventions.

Although several studies have reported utility values associated with overweight and obesity in adults [22,23], we believe ours is the first study to report utility values for overweight/obese children and adolescents. To date, cost-effectiveness analyses of pediatric interventions have focused on averting adult obesity [24,25], but having an estimate of the decrement in health utility associated with child obesity extends the ability of such analyses to account for childhood-specific benefits of the interventions as well. Domains of health may have different relative weights for children as compared with adults and result in a different level of overall quality of life for the same health state in patients of different ages. Since there has been little data on health utilities in pediatric health states to suggest where differences may be greatest, more study of health utilities in pediatric health states is needed [26]. This study makes an important contribution in the area of understanding quality of life for overweight/obese children.

A main finding of our study was that overweight/obese children have a health utility value 0.04 points lower than healthy weight children. Differences of 0.05 on the HUI are thought to be clinically meaningful, and some investigators report that differences as small as 0.03 may be important [27-29]. The difference in utility we noted between healthy weight and overweight/obese children is similar to the results of Livingston et al [30] who calculated utilities from health status questions in the National Health Information Survey, and found that the utility of overweight adults was 0.04 lower than normal weight adults; the utility of obese adults was 0.07 lower than healthy weight adults. Our estimate of the overall health utility for overweight/obese children and adolescents was 0.78, lower than the utility of obese adult women reported by Roux et al [12], but similar to two other studies in adults [25,31]. Thus, although our results lack the precision needed to meet statistical significance, our point estimate of the difference in utility between healthy weight and overall/obese children suggests that there is likely to be a measurable and clinically important decrement in utility value associated with overweight status in children and adolescents that is similar in magnitude to that seen in adults.

Of note in our study, parent ratings of their overweight/obese children's utility was higher than the children's own rating, whereas for normal weight children, parents' and children's own ratings were similar. Several studies suggest that parents frequently misperceive their children's weight status, tending to classify their overweight or obese children as being of healthy weight [32,33]. Parents may also be less aware than their children of physical and psychological consequences of overweight/obesity. In future research on health state preferences associated with weight status, investigators should consider both the child's and the parent's perspective.

We could identify only one prior study that used the HUI in the setting of pediatric obesity research. The APPLE Project [34], a child obesity prevention program in New Zealand, administered the HUI to healthy school children at the beginning and at the end of their intervention, and did not observe a measurable change in HUI scores despite a decrease in BMI that occurred over the course of the intervention. Generic measures of health status, such as the HUI, may be less sensitive to the effects of weight status on health than obesity-specific instruments, such as the one developed by Mathias et al [22] and the Impact of Weight on Quality of Life-Lite (IWQOL-lite) [23], or may measure different effects of the disease state on health [35]. Those obesity-specific instruments were developed for and validated in adults; currently, no obesity-specific preference based measure exists for children. In the future, researchers should consider adapting adult obesity-specific measures to the pediatric population, or developing new tools for use in pediatric obesity. Alternatively, utilities can be measured directly using methods such as time-tradeoff and standard gamble, although the methodologic issues related to using those techniques in children and adolescents bear careful consideration and would require proxy respondents for children younger than 12 years old [36].

Generalizability of our findings may be limited by our use of a convenience sample, which was drawn from primary care and obesity clinics at an urban academic children's hospital. Additionally, the relatively small number of obese children in our sample limits our ability to compare utilities between overweight and obese children. A strength is that we used a well-validated instrument to measure health status and calculate health utilities, a method which is substantially less time-intensive and costly than direct measurement. Our results also allow the calculation of the sample size needed to detect the difference in health utility between normal weight and overweight/obese children and adolescents, approximately 182 participants in each group.

## Conclusion

Ours is the first study to report health utility values associated with childhood overweight and obesity. Using the HUI, we found that overweight and obese children and adolescents trended towards lower utility values that healthy-weight children. We believe that by providing a quantitative estimate of the decrease in health status associated with overweight and obesity in children and the degree of uncertainty around that estimate, our findings will be of particular use to researchers assessing cost-effectiveness of childhood obesity prevention and treatment interventions.

## Abbreviations

HRQOL: health related quality of life; HUI: Health Utilities Index

## Competing interests

The authors declare that they have no competing interests.

## Authors' contributions

MBB, JAFZ, and LP conceptualized and designed the study. KMR and JT collected the data. MBB analyzed the data. MBB, JAFZ, and LP interpreted the results. MBB drafted the manuscript; all authors read and approved the final version of the manuscript.

## Pre-publication history

The pre-publication history for this paper can be accessed here:

http://www.biomedcentral.com/1471-2431/11/12/prepub
